# Influence of CuO on the performance of conducting polymer matrix for screen-printed humidity sensing applications

**DOI:** 10.1007/s00604-026-07897-9

**Published:** 2026-02-17

**Authors:** B. S. Manjunatha, Shilpa Shetty, Mohammad Saquib, Suma A Rao, Ramakrishna Nayak, Vinod Kamath, M. Selvakumar

**Affiliations:** 1 Department of Chemistry, Manipal Institute of Technology, Manipal Academy of Higher Education, Manipal, Karnataka 576104 India; 2 Department of Humanities and Management, Manipal Institute of Technology, Manipal Academy of Higher Education, Manipal, Karnataka 576104 India

**Keywords:** Conducting polymers, Metal oxide composites, Flexible electronics, Printed sensors, Humidity sensing, Screen-printing technique

## Abstract

A flexible polyethylene terephthalate (PET) substrate was employed for the fabrication of a resistive-type humidity sensor using screen-printed polypyrrole (PPy)/CuO nanocomposite conductive inks. A series of conducting polymer-metal oxide composite-based sensors with varying compositions was prepared, and their humidity-sensing performances were systematically evaluated. The structural and morphological features of the composites were characterized using scanning electron microscopy (SEM), Fourier-transform infrared spectroscopy (FTIR), and X-ray diffraction (XRD). The sensing behaviour of the printed devices was investigated over a relative humidity (RH) range 22–97% at room temperature, focusing on parameters such as relative resistance, sensitivity, dynamic response, and hysteresis. Enhanced sensitivity was achieved with the incorporation of CuO into PPy. The superior sensing performance is attributed to the combined structural characteristics and hydrophilic nature of CuO. The PPy/CuO nanocomposite with a 1:1 weight ratio, prepared via mechanical mixing and screen printing, exhibited the highest sensitivity over a broad humidity range (22–97% RH), along with fast response/recovery times (~ 50/60 s) and negligible hysteresis.

**Graphical Abstract:**

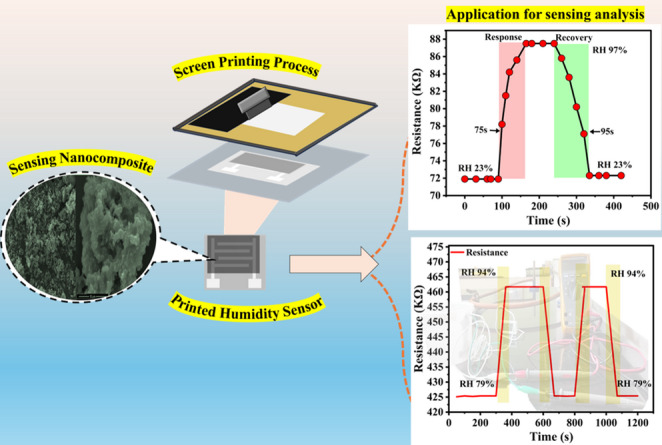

**Supplementary Information:**

The online version contains supplementary material available at 10.1007/s00604-026-07897-9.

## Introduction

Humidity defined as the amount of water vapour present in the atmosphere [1], plays an important role in many areas including agriculture, healthcare [2–4] and food storage [5, 6] where controlling and maintaining optimal environmental conditions is required and the relative humidity is monitored by using humidity sensors [7]. Conventional humidity sensors fabricated by metal oxides and ceramic materials are associated from several drawbacks such as high fabrication cost, complex processing methods relatively high-power utilization [8, 9]. Conductive polymers have come up as promising alternative to address these limitations and several studies highlighted their utilization for sensor applications [10–12]. Among the different kinds of sensors, the resistive type has gathered more attention due to simple fabrication and economy [13, 14]. Despite this advantage there are some performance features like response and recovery times, dynamic range and measurement accuracy still needed more elaborate attention [15, 16]. Polypyrrole (PPy) is one of the materials widely utilizes for moisture sensing owing to its high environmental stability, facile synthesis, distinctive electrical conductivity and easily integrated into diverse sensing platforms [17, 18]. PPy synthesised through chemical or electrochemical methods in aqueous and non-aqueous media offering significant flexibility in the fabrication [19]. In addition to pristine PPy many studies have demonstrated the improved performances of the PPy -metal oxide composites as sensing materials [20, 21]. For instance, Shukla et al. demonstrated the humidity sensing performances of a PPy-ZnO composite synthesised through in situ polymerization and monitored the sensing character under controlled relative humidity using saturated salt solutions [22]. Pan et al. developed a magnesium oxide (MgO) based semiconductor humidity sensor through microarc oxidation technique. Pathan et al. explore resistive type humidity sensor of ZnO/CuO nanostructures and demonstrated their enhanced sensing performances [23]. Pathan et al. explore resistive type humidity sensor of ZnO/CuO nanostructures and demonstrated their enhanced sensing performances [24]. In recent report, B. Chetan et al. showed that the incorporation of TaO into polypyrrole increased the porosity and water absorption efficiency resulting in a remarkable sensing performance compared to pure polypyrrole [25]. A. Yussuf et al. synthesised PPy via oxidative polymerization at room temperature using ammonium persulfate and ferric chloride with sodium lauryl sulphate as a surfactant. Their result showed a significant reduction in the resistance when ferric chloride was used in the synthesis and achieved improved stability and reduced electrical resistance when the oxidant-to-monomer ratio was 2:1 [26]. Najjar et al. synthesised hybrid polypyrrole with different loading of ZnO nanoparticles through oxidative polymerization process. A high sensitivity of 98% was attributed to the formation of p-n junction developed by the covalent interactions between the PPy chains and the nanoparticles which promote the more efficient charge transfer between the metal oxide and the polymer [27]. Duan et al. developed a humidity sensor using carbon ink incorporated carbon nanoparticles and evaluated its sensing characteristics at 20 °C. They predicted a positive humidity response whereby the resistance increased with that of relative humidity [28]. D wang et al. used PVA/MXene composite nanofibers film to fabricate a self - powered sensor in which a MoSe_2_ piezoelectric nanogenerator (PENG) acted as a driving force. By converting the mechanical energy to electrical energy this device sensed the humidity. The PENG developed a distinct output voltage by reaping energy from the different parts of the body and exposed its application as a suitable material for the wearable sensors. A low hysteresis of 1.8% and a response/recovery time of 0.9/6.3 s were promising feature of the device [29]. W Liu et al. developed an ultra-thin film humidity sensor by electrostatic self-assembly method by using TiO_2_ quantum dots/silica composites. This sensor showed a multifunctional capability like human body detection, humidity alarm system and voice recognition applications [30]. From these studies one can conclude the importance of conducting polymer and their composites as a promising material for sensor applications. Copper (II) oxide a p-type semiconducting transition metal oxide has gathered considerable attention particularly catalyst and gas sensors. A metal-organic frame-work (MOF) derived CuO has gained much interest recently because of its tuneable chemical properties and well-defined porous structure provide improves sensing performances [30, 31]. Moreover, this material is of particularly interest because of its redox activity associated with Cu^2+^/Cu^+^ couple and the presence of abundant reactive sites. These features promote the effective water adsorption via physisorption and chemisorption processes. When integrated with PPy, CuO forms a synergistic composite in which CuO aids water molecule interaction while PPy enhance the charge transport and signal amplification, resulting in improved sensitivity, selectivity and response time [32, 33]. Printing electronics have emerged as a strong alternative to the traditional silicon-based electronics especially in flexible, wearable and disposable applications [34]. Among the available fabrication approaches screen printing method is more encouraging due to its simplicity, low cast and suitability for larger scale production. In this method, the ink formulation comprises the active material blended with additives and suitable solvents [35]. These above components play a important role in preventing the space separation while also improving design resolution, stability and adhesion. The desired structural, functional characteristics and the printability of the formulated ink is governed by the rheological properties. This highlights the need of the present work in the study of suitable materials and ink formulation for the sensors. The present work comprises of preparation of conductive ink using PPy/CuO composite by taking different weight ratio of the components. Cellulose acetate propionate (CAP) was used as the binder and a highly polar aprotic solvent dimethylformamide (DMF) with surface tension of 37.1 mN/m acted as the dispersion medium. The DMF facilitated the development of structural integrity, promoted interchain interaction within PPy and to disperse the composite particles homogeneously. A uniform film formation can be expected due to its high boiling point and the controlled evaporation. The CAP ensured the additional film uniformity and the mechanical stability during the printing technique. By screen printing process the humidity sensors were fabricated by depositing the optimised composite conductive ink onto flexible PET substrates with silver electrodes served as current collectors. Properties like viscosity, surface tension and contact angle of the formulated inks were systematically optimized to achieve superior film quality and printability. The performances of the printed humidity sensor devices were monitored under controlled relative humidity in the range 23% to 97% at room temperature using saturated salt solutions to maintain the accurate humidity values.

## Experimental section

### Materials

Pyrrole [C_4_H_4_NH] 99% purity (which was refrigerated at 4 °C until used), Ferric chloride hexahydrate, 99% purity [FeCl_3_.6H_2_O], and Sodium lauryl sulphate (SLS) were procured from LOBA. Copper (II) oxide [CuO], Dimethylformamide (DMF), cellulose acetate propionate (CAP), methanol, magnesium chloride, magnesium nitrate, cupric chloride, potassium chloride, and potassium sulfate were procured from LOBA Chemicals. All reagents were of analytical grade and used as received without further purification. Electrical resistance and current measurements were carried out using a Fluke 179 True RMS Multimeter.

### Ferric chloride-mediated chemical synthesis of polypyrrole

**Scheme 1:**
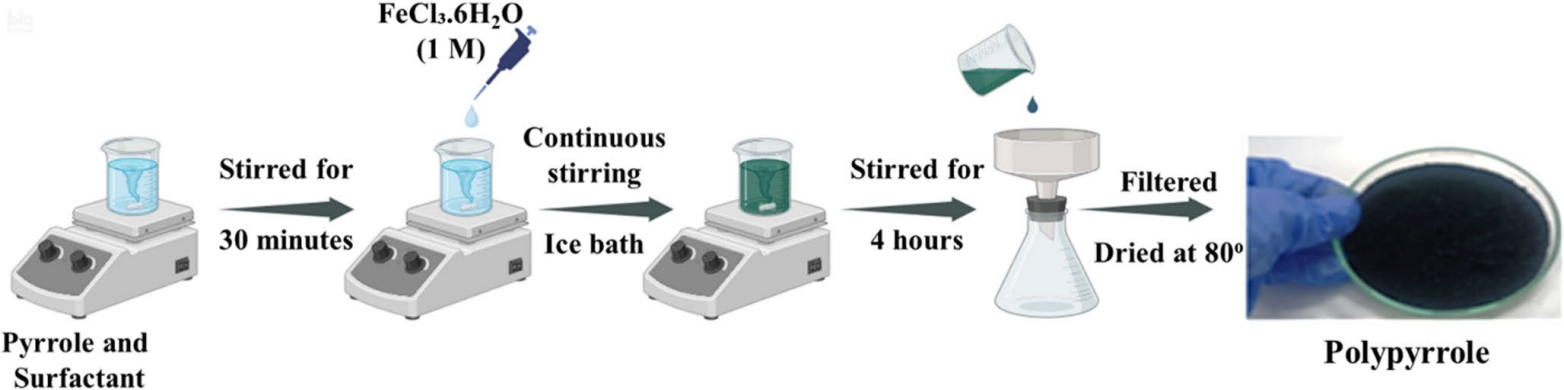
Synthesis of polypyrrole via chemical oxidative polymerization of pyrrole and surfactant using FeCl_3_·6H_2_O as oxidant

Polypyrrole (PPy) was synthesized through the chemical oxidative polymerization of pyrrole monomer using ferric chloride as the oxidizing agent. Initially, a polymer-surfactant solution was prepared by dissolving pyrrole monomer (0.5 M) and sodium lauryl sulphate (SLS, 0.02 M) in 50 mL of distilled water. The mixture was stirred thoroughly to ensure homogeneity for 30 min. Separately, an aqueous solution of FeCl_3_.6H_2_O (1 M) was prepared in 50 mL of distilled water and added dropwise to the monomer-surfactant solution under continuous stirring at a controlled temperature of 20 °C. The polymerization reaction was allowed to proceed for 4 h under constant stirring. Upon completion of the reaction, the resulting black polymeric precipitate was collected and washed repeatedly with deionized water followed by methanol to remove unreacted monomer, oxidant, and surfactant residues. The purified polypyrrole product was then dried in an oven at 80 °C for 4 h to obtain the final dry powder [22, 23, 36]. Scheme 1 shows the synthetic approach for the polypyrrole and nanocomposite that is employed for further formulation and fabrication of humidity sensor.

### Conductive ink formulation for humidity sensor fabrication

The synthesized polypyrrole (PPy) was blended with copper (II) oxide (CuO) in different weight ratios (1:1, 2:1, 3:1, 1:2, and 1:3) through mechanical mixing for 60 min to obtain uniform PPy/CuO composites. For conductive ink preparation, the composites were dispersed in dimethylformamide (DMF, 80 wt%) with cellulose acetate propionate (CAP, 20 wt%) employed as a polymeric binder. The mixtures were stirred for 1 h to ensure homogeneous dispersion. The final ink formulation contained 10 wt% of the composite material, which provided rheological properties well-suited for screen-printing applications [24]. Table 1 shows the abbreviations used hereafter in this article to denote the respective polymer/metal oxide nanocomposites.

**Table 1:** Denotations of different weight ratios of polypyrrole (PPy)/copper (II) oxide (CuO) nanocomposites

Sensor denotation	PPy: CuO weight ratio
P1	1 : 0
PC31	3 : 1
PC21	2 : 1
PC11	1 : 1
PC12	1 : 2
PC13	1 : 3

### Fabrication of humidity sensors

**Scheme 2:**
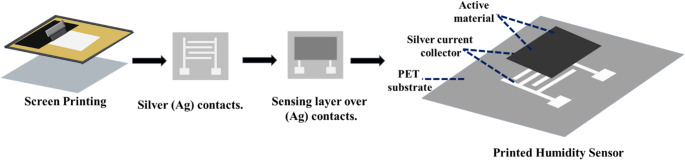
Fabrication of a humidity sensor on a PET substrate involving screen printing of silver contacts, deposition of the sensing layer, and subsequent device assembly

Humidity sensors were fabricated by screen-printing a silver electrode pattern onto a polyethylene terephthalate (PET) substrate. The prepared PPy/CuO conductive ink was then printed over the silver electrodes. Film thickness and uniformity were achieved through sequential deposition of multiple layers, with each layer dried prior to the next; a total of three overprints were applied in this study. Screen printing was performed using a polyester screen with a mesh density of 120 threads cm^−1^ in both directions and a polyurethane squeegee under controlled manual pressure and printing speed to ensure uniform ink transfer. Each printed layer was thermally dried at 50 °C for 30 s, followed by cooling at room temperature for 30 s before subsequent printing. This procedure was consistently applied to both the silver current collector and the sensing layer to ensure uniform thickness and strong interlayer adhesion. The same procedure was followed for all PPy/CuO composite formulations. After printing, the sensors were thermally treated at 70–80 °C for 4 h and stored in a vacuum desiccator to maintain their structural and functional integrity before characterization [25, 26, 37]. Scheme 2 shows the fabrication and printing process of a humidity sensor over a flexible PET substrate.

## Humidity sensing analysis

The humidity-sensing performance was evaluated using devices fabricated with different compositions of the polypyrrole/copper oxide (PPy/CuO) conductive inks. The fabrication process began by applying pressure to uniformly disperse the conductive silver ink through the screen mesh onto a PET substrate, forming the current collector. The formulated PPy/CuO inks were then screen-printed onto the pre-deposited silver electrodes. After coating, the films were inspected for cracks, and the number of overprints was kept constant to minimize variations in active material thickness. The fabricated devices were subsequently thermally treated in a hot air oven at 60 °C for 1 h. Humidity levels ranging from 22% to 97% were generated using saturated salt solutions of potassium acetate, magnesium chloride, magnesium nitrate, cupric chloride, potassium chloride, potassium nitrate, and potassium sulfate, each equilibrated for 12 h [30]. All measurements were conducted at room temperature (25 ± 1 °C). The relative humidity values produced by the saturated salt solutions were verified using a commercial hygrometer before the measurements.

The sensors were suspended 2 cm above the solution surface, and their electrical response was recorded using a digital multimeter [26],]. Device response at different relative humidity levels was monitored, and sensitivity was calculated according to **Eq. (1)**.

1 $$\:S_{RH}=\left[\left(\:\frac{R_i\:-{\:R}_{RH}}{R_i}\right)\:\times\:100\right]$$

*R*_*i*_ indicates initial resistance, *R*_*RH*_ refers to resistance at a specific RH value, and *S*_*RH*_ denotes the sensitivity at the same.

Hysteresis is defined as the difference in the sensor response during the adsorption and desorption cycles. The average percentage hysteresis of the fabricated humidity sensors is calculated using the following relation:

2 $$Full-scale\:value=R_{max}-R_{min}$$

3 $$H\;average\;\left(H_{avg}\right)=\frac{\sum\:Rd-Ra}n$$

where $\:"n"$ is the number of values measured, and $\:"Ra"\:$ and $\:"Rd"\:$ represent the sensor resistance during adsorption and desorption, respectively.

4 $$\:Average\:percentage\:hysteresis\:\left(H_{avg\%}\right)=\frac{H_{avg}}{Full\;scale\;value}\times100$$

5 $$Maximum\:percentage\:hysteresis=\frac{H_{max}}{Full\:scale\:value}\times100\:$$

6 $$Minimum\:percentage\:hysteresis\:=\frac{H_{min}}{Full\:scale\:value}\times100$$

## Structural and physicochemical characterization

The comprehensive spectroscopic and physicochemical characterizations were performed on pristine polypyrrole, its composites, and the corresponding conductive ink formulations. Fourier-transform infrared (FTIR) spectra were recorded using a Shimadzu IR Spirit spectrometer to identify functional groups and probe molecular interactions. Surface morphology and structural features were analysed through scanning electron microscopy (SEM) and X-ray diffraction (XRD) using a Rigaku Miniflex 600 diffractometer equipped with Cu Kα radiation. The rheological behaviour of the conductive inks, including viscosity profiles under varying shear rates, was evaluated with a high-precision modular rheometer using Anton Paar’s RheoCompass software. Thixotropic properties were assessed via a three-interval thixotropy test (3ITT). Additionally, wettability and surface tension characteristics were measured by depositing the inks directly onto substrates using an Ossila goniometer. The thickness of the sensor devices were evaluated by using a Mitutoyo Absolute digital thickness gauge.

## Result and discussion

### FTIR analysis

**Fig. 1:**
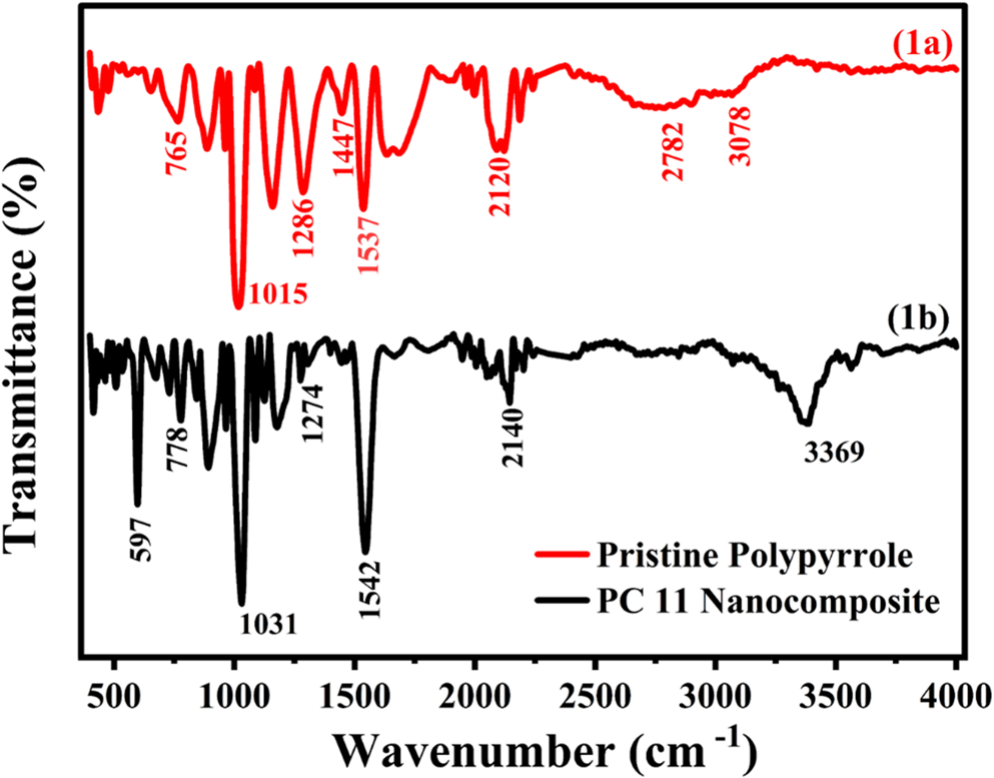
FTIR spectrum of pristine PPy and PPy/CuO nanocomposite

The FTIR (Fourier-Transform Infrared Spectroscopy) spectrum of pristine polypyrrole (PPy), shown in Fig. 1a, exhibits characteristic absorption bands. Absorption bands at 2782 and 3078 cm^− 1^ correspond to symmetric C-H stretching vibrations. The band at 1537 cm^− 1^ corresponds to C-C and C = C stretching of polypyrrole. The band at 1447 and 1286 cm^− 1^ is assigned to C-C stretching or ring vibration within the pyrrole and C-N stretching coupled with C-H deformation respectively. The bands 1015 and 765 cm^− 1^ correspond to the in-plane and out-of-plane C–H bending modes [6, 38]. The band at 1286 cm^− 1^ is assigned to C-N stretching coupled with C-H deformation, and the peaks at 1015 and 765 cm^− 1^ correspond to the in-plane and out-of-plane C–H bending modes [39, 40]. The FTIR spectrum of the PPy/CuO composites **(**Fig. 1b**)** retained the characteristic absorption bands of pristine polypyrrole, confirming the presence of the polymer matrix. Notable variations, including peak shifts, band broadening, and intensity changes, were observed, indicative of structural perturbations and potential electronic interactions between PPy and CuO. The successful incorporation of copper (II) oxide was confirmed by the appearance of a new intense band at 597 cm^− 1^, corresponding to the Cu-O stretching vibration [41]. Furthermore, the detailed FTIR peak assignments and their comparative wavenumber analysis for the pristine material and the nanocomposite are provided in Tables **S1** and **S2** in the Supporting Information.

### XRD analysis

**Fig. 2:**
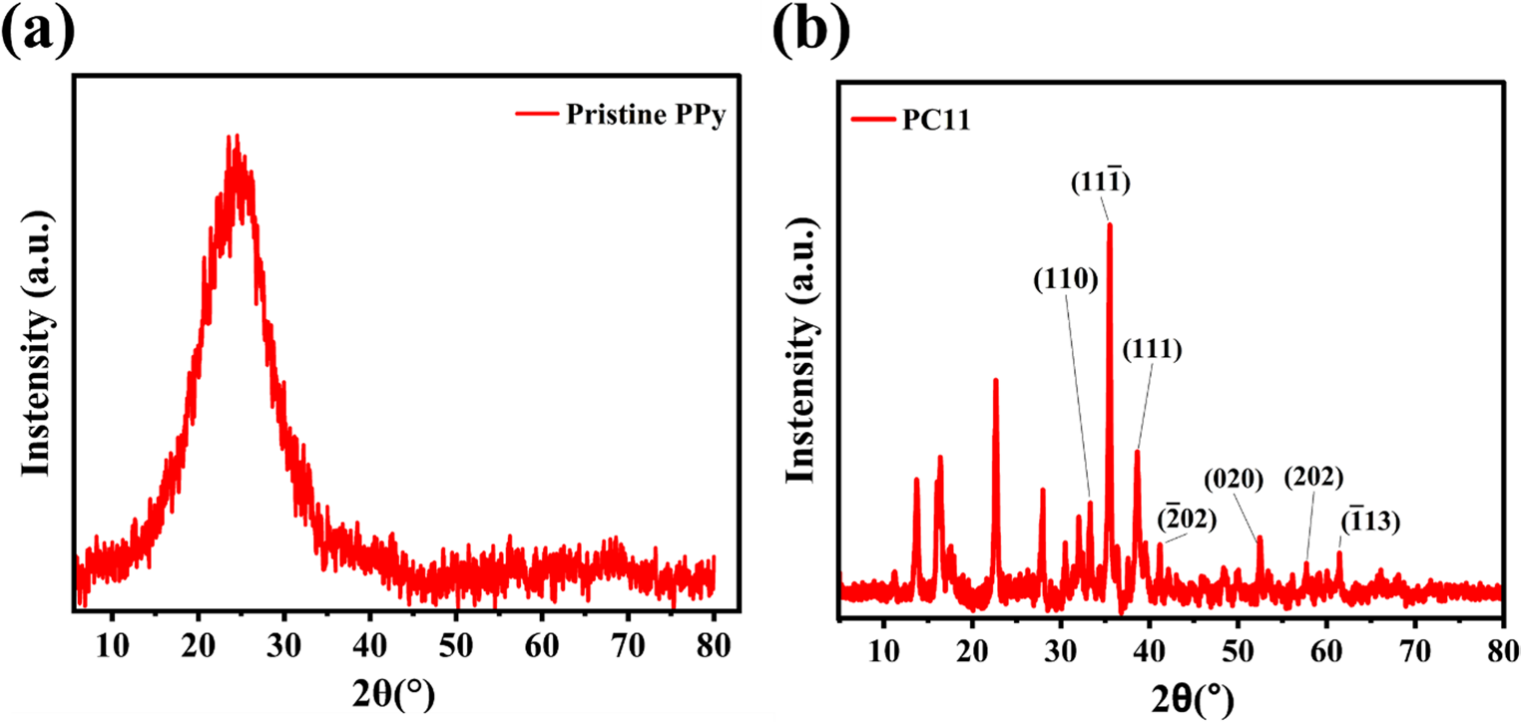
(**a**, **b**). XRD analysis of (**a**) pristine polypyrrole and (**b**) polypyrrole/copper oxide nanocomposite

The structural features of pristine polypyrrole (PPy) and its CuO composite were examined using X-ray diffraction (XRD), as shown in Fig. 2a. The XRD pattern of pristine PPy exhibits a broad diffraction peak centered around 20–25°, indicating its amorphous or poorly crystalline nature, characteristic of intrinsically conducting polymers [42]. This broad hump arises from π–π stacking interactions between conjugated polymer chains, reflecting interchain spacing without long-range order. In contrast, the PPy/CuO composite, as shown in Fig. 2b, displays multiple distinct diffraction peaks superimposed on the broad PPy background, corresponding to the crystalline CuO phases within the amorphous polymer matrix. Peaks at 2θ values of 32.5°, 35.5°, 38.7°, 48.5°, 52.4°, 57.3°, and 61.4° were assigned to the ($\:110$), ($\:\overline{1}11$), ($\:111$), ($\:\overline{2}02$), ($\:020$), ($\:202$), and ($\:\overline{1}13$) planes, in agreement with JCPDS 48–1548. The coexistence of sharp crystalline and broad amorphous peaks confirms the heterogeneous structure of the composite and the successful incorporation of crystalline CuO nanoparticles into the polypyrrole matrix [43]. Furthermore, the crystallite size was calculated using the Debye–Scherrer equation (**Eq. 7**). For the optimized nanocomposite PC11, the calculated crystallite size was approximately 290 nm. This optimized nanocomposite exhibited enhanced adsorption/desorption capability, outperforming the other nanocomposites, which can be attributed to its favourable crystalline characteristics.

7 $$\:D=\frac{K\lambda\:}{\beta\:cos\theta\:}$$

Where the Scherrer Eq. (7), $\:"D"\:$represents the average crystallite size (nm), $\:"K"\:$is the shape factor with a typical value of 0.9, $\:"\lambda\:"$ denotes the X-ray wavelength, $\:"\beta\:"\:$corresponds to the full width at half maximum (FWHM) of the diffraction peak measured in radians, and $\:"\theta\:"\:$is the Bragg diffraction angle.

### FESEM analysis

**Fig. 3:**
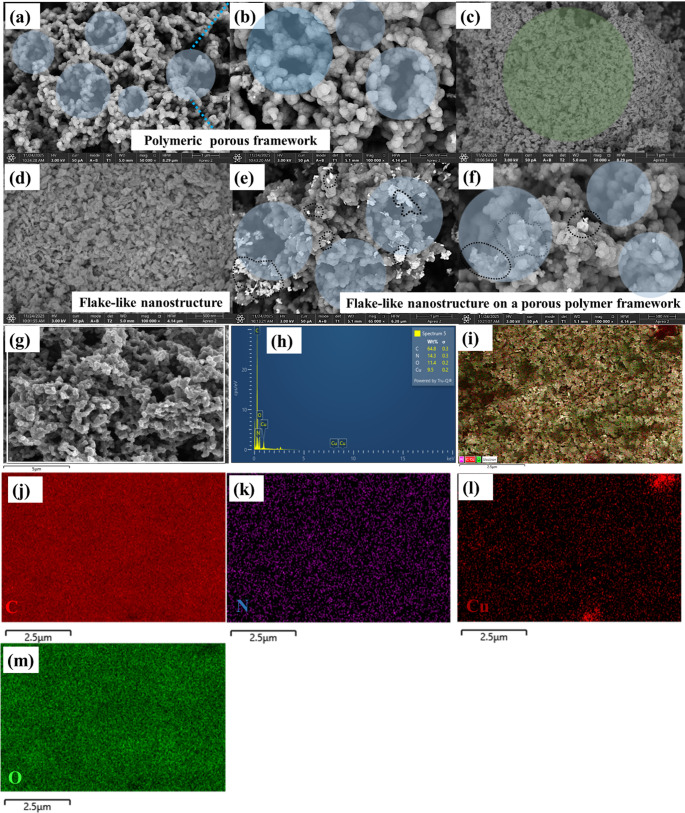
FESEM images of (**a**, **b**) (PPy) nanoparticles at different magnifications, showing a porous polymeric framework, and (**c**, **d**) the PPy/CuO (PC11) nanocomposite with a flake-like morphology. Higher-magnification images (**e**, **f**) highlight the distribution of CuO nanostructures on the PPy framework. Image (**g**) shows the overall surface morphology. The EDS spectrum (**h**) confirms the presence of C, N, O, and Cu, while elemental mapping images (**i**-**m**) demonstrate the uniform elemental distribution within the nanocomposite

The Fig. 3 illustrates the surface morphology of the synthesised polypyrrole and the metal oxide composite. The Field emission scanning electron microscopy (FESEM) analysis confirmed an irregular granular morphology for the pure polypyrrole which is the characteristic of the material synthesised via chemical oxidation process (Fig. 3(a) and 3(b)). The granular morphology of the pristine polypyrrole is normally attributed to the development of solid-state nuclei during the initial polymerization process followed by subsequent adsorption of the oligomers onto these nuclei. The granular morphology of the pristine polypyrrole is normally attributed to the development of solid state nuclei during the initial polymerization process followed by subsequent adsorption of the oligomers onto these nuclei [44, 45]. The reaction condition and the surface concentration exhibit a strong influence in determining the polymer morphology. Surfactant concentration level exceeding 100 mM result in irregular granular size while elevated temperature leads to a spongy, porous structure and lower temperature produce a more compact and denser morphology. Accordingly, polypyrrole synthesised by chemical oxidation process in presence of FeCl_3_ and SLS shows morphology variations depending on the concentration level of surfactant and the monomer. These variations arise from alteration in the nucleation and growth dynamics during the polymerization which in turn govern the structural characteristics of the resulting polymer. The SEM image of the PC11 nanocomposite as illustrated in Fig. 3(c) and 3(d), display a porous and heterogeneous morphology resulting from the successful incorporation of the copper oxide into the polypyrrole matrix. In contrast to the relatively uniform granular morphology of pristine polypyrrole as shown in Fig. 3(a) and 3(b), the PC11 nanocomposite shows agglomerated cluster of PPy/CuO nanoparticles with enhanced surface roughness and interstitial voids. This porous and irregular architecture is indicative of the effective incorporation of the CuO within the polypyrrole matrix, resulting to an enlarged surface area and provide additional active sites that ease the water molecule adsorption as shown in Fig. 3(e) and 3(f). These structural features are beneficial for humidity sensing as they can promote rapid diffusion of the water vapour and improve the sensitivity of the fabricated devices [46, 47]. To further comprehend the material high resolution energy dispersive X ray spectroscopy along with elemental mapping was performed on the optimized nanocomposite to explicate its elemental composition and special distribution. The EDX spectrum shown in Fig. 3(h) illustrate the presence of all the constituent elements namely carbon, nitrogen, copper and oxygen with their corresponding atomic weight% confirming the effective incorporation of CuO into the polypyrrole matrix. Additionally, elemental mapping analysis (Fig. 3(i-m)) elucidates a uniform and homogeneous distribution of all elements across the nanocomposite surface. Figure 3**(i)** demonstrates the high-resolution FESEM image used for mapping while the corresponding colour coded elemental maps show the spatial dispersion of carbon (dark red), nitrogen (purple), copper (red) and oxygen (green). The homogeneous element distribution indicates increased interactions between PPy and CuO, which are more advantage for the effective charge transport and electrochemical performance.

## Rheological analysis

### Viscosity analysis

**Fig. 4:**
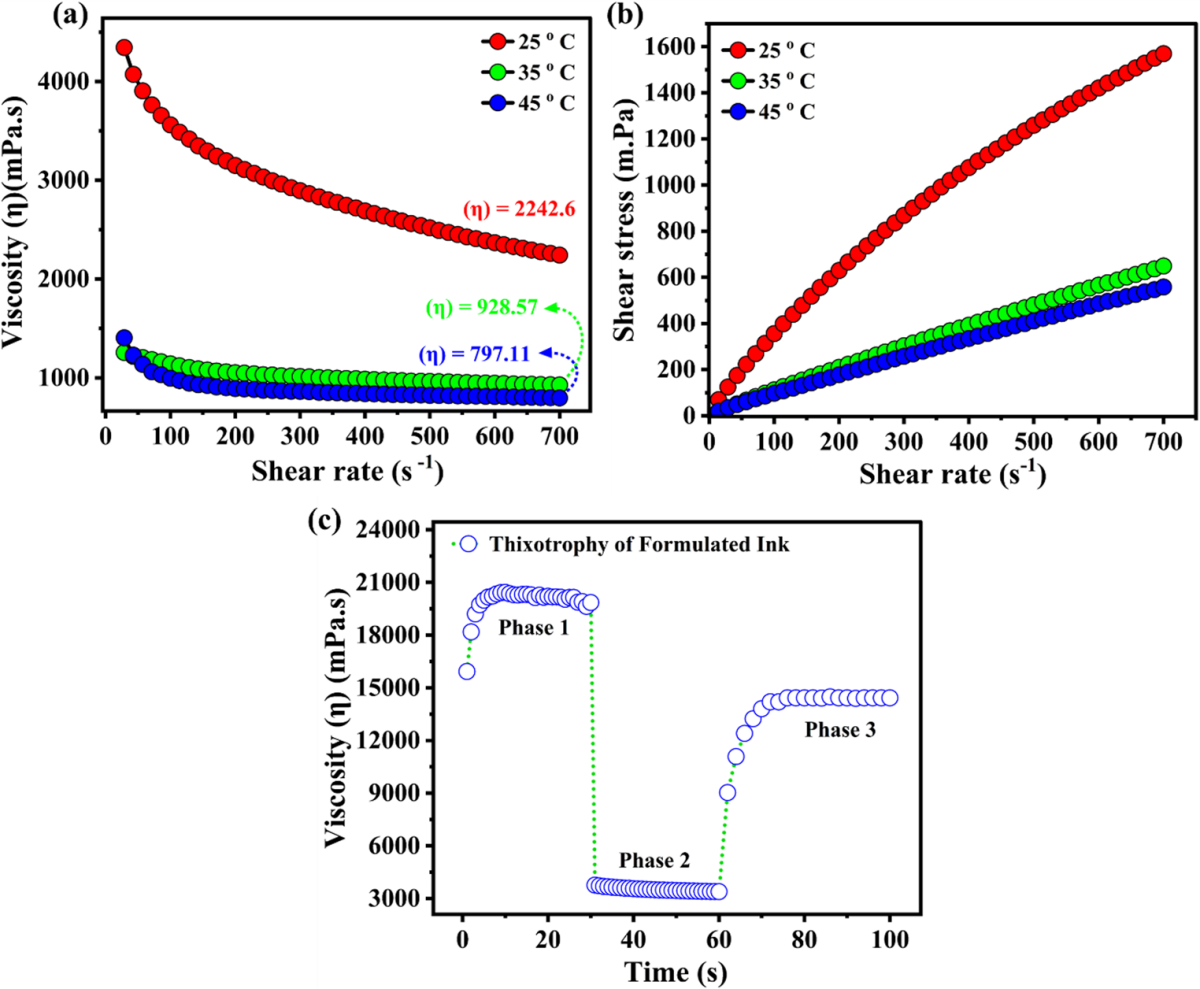
Rheological characteristics of the formulated ink, (**a**) variation of viscosity with shear rate, showing shear-thinning behaviour, (**b**) relationship between shear stress and shear rate confirming pseudoplastic fluid characteristics, and (**c**) thixotropic response illustrating reversible viscosity recovery under cyclic shear

The rheological behaviour of the polypyrrole-copper oxide (PPy-CuO) composite conductive ink were evaluated at 25 °C, 35 °C and 45 °C. Figure 4(a) exhibits a pronounced shear thinning behaviour at all the temperatures, as evidenced by continuous decrease in viscosity with the increase in shear rate. The continuous disentanglement and alignment of the polymer chains and particle network under applied shear results in this non-Newtonian behaviour, thereby promoting easier flow through the printing mesh. At 25 °C the ink showed the highest viscosity, declining from 4500mPa.s at low shear rate to approximately 2500 mPa.s at higher shear rate. A sharp decrease in viscosity with values levelling off in the range 900–1000 mPa.s was observed at elevated temperature of 35 °C and 45 °C under high shear rates. This temperature dependant decline in viscosity is as a result of increased polymer chain mobility and poor intermolecular interaction which collectively improve flow characteristics. Such temperature dependant rheological behaviour is more desirable for printing applications, as ink retains sufficient viscosity at rest to show stability while exhibiting reduced flow resistance under shear, promoting efficient deposition during the screen- printing process. The shear stress versus shear rate profile as presented in Fig. 4(b). characterize the measured rheological behaviour of the conductive polymer composite ink. The non-Newtonian shear thinning nature of the formulated ink is confirmed by the progressively decreasing slope on a logarithmic scale. This indicates the viscosity decrease with increasing shear rate thereby facilitating improved flow under the applied stress. Subsequent measurement at higher temperature of 35 °C and 45 °C demonstrate a marked reduction in shear stress at a given shear rate which can be ascribed to the decrease in viscosity. This temperature dependant rheological behaviour confirms the suitability of the ink for printing applications where ease of uniform spreading and consistency under mechanical stress are crucial. Figure 4(c) illustrate the time dependant viscosity response under varying stress conditions, explicating vital insight into its rheological stability. At reduced shear rate the ink shows stable viscosity because of the network or well - developed internal microstructure that prevents the flow, thereby increasing the stability during the storage. Such a structured network typically results from the aggregation of polymer chains and the dispersed CuO particles. The second interval reveals a significant drop in the viscosity at a higher shear rate. This reflects the breakdown of the internal structure under stress, a shear-thinning behaviour which is vital for the ink’s smooth flow during printing. The decrease in viscosity during shear rate allowed the conductive ink to pass through the screen mesh without clogging during printing. The viscosity is regained to its original level as the shear rate is reduced in the third interval. But the incomplete return to its initial value confirms the thixotropic nature of the ink and the partial recovery of the structure. The increased viscosity at rest, the low viscosity under shear, and the partial recovery post shear are the desirable rheological profiles for the screen-printing ink. These properties guaranteed that the formulated conductive inks remain stable during storage, flow easily under printing, and prevent spreading or bleeding after deposition.

### Contact angle and surface tension of formulated ink

**Fig. 5:**
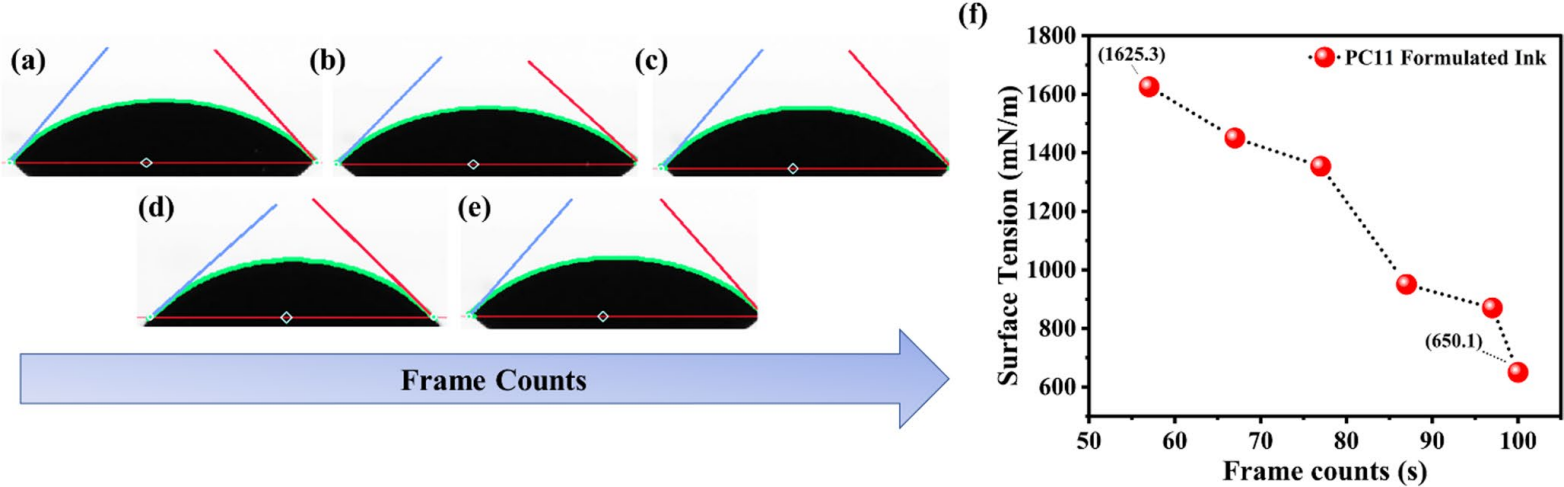
Contact angle images of “PC11” nanocomposite formulated ink captured at different frame counts, (**a**) 55.83º (57th frame), (**b**) 49.48º (67th frame), (**c**) 48.52º (87th frame), (**d**) 43.03º (100th frame), and (**e**) further reduction with time, showing the wetting behaviour of the ink on the substrate, (**f**) Corresponding surface tension profile of the PC11 formulated ink as a function of frame counts, decreasing from 1625.3 mN/m to 650.1 mN/m

Figure 5 (a-e) shows the contact angle of the formulated ink evaluated using an Ossila goniometer. The contact angle plays a critical role in governing the printability of an ink as it directly refers its wetting character on the substrate surface. A lower contact angle suggests a good wetting behaviour which facilitates strong adhesion and uniform spreading of the ink. Hence the determined contact angle approves the suitability of the ink for printing process as it reveals amicable interaction with the substrate surface [27]. The plot showed that with an increase in frame count, corresponding to the progression of time the contact angle gradually decreases and reaching approximately 43º by frame 100. This decline signifies a transition from a partially wetting to superior wetting behaviour, reflecting more enhanced spreading of the formulated ink over the substrate surface. This time dependent decrease in contact angle is due to the dynamic rearrangement and redistribution of the surface-active components in the ink formulation as shown in the Fig. 5(f). Specifically, the diffusion and orientation of the surfactant molecules or polar functional groups towards ink-air and ink-substrate interface can enhance the wetting over time. In conjunction with the surface tension results, the decline in contact angle further substantiates the dynamic wetting behaviour of the polypyrrole-copper oxide composite ink, revealing its aptness for the high resolution and consistent pattern formation through screen printing. In screen printing preserving an optimal surface tension is crucial to obtain consistent ink transfer, increased substrate wetting and the development of well-defined patterns. An excessive surface tension doesn’t facilitate the spreading ability of the ink over the substrate leading to poor wetting characteristics. Such conditions can lead to issues like beading, incomplete coverage, and poor interfacial adhesion which ultimately reduce the pattern uniformity, resolution and electrical performances in the case of functional ink. On the other hand, PPy/CuO composite inks with surface tension optimized to match the printing conditions exhibits superior spreading pattern and enhanced interfacial adhesion. This optimization ensure the ink to form uniform and continuous patterns with minimal defects which is crucial for ensuring the reliability and resolution required for the consistent performances of the printed sensor devices [48, 49].

### Response characteristics of the humidity sensor

**Fig. 6:**
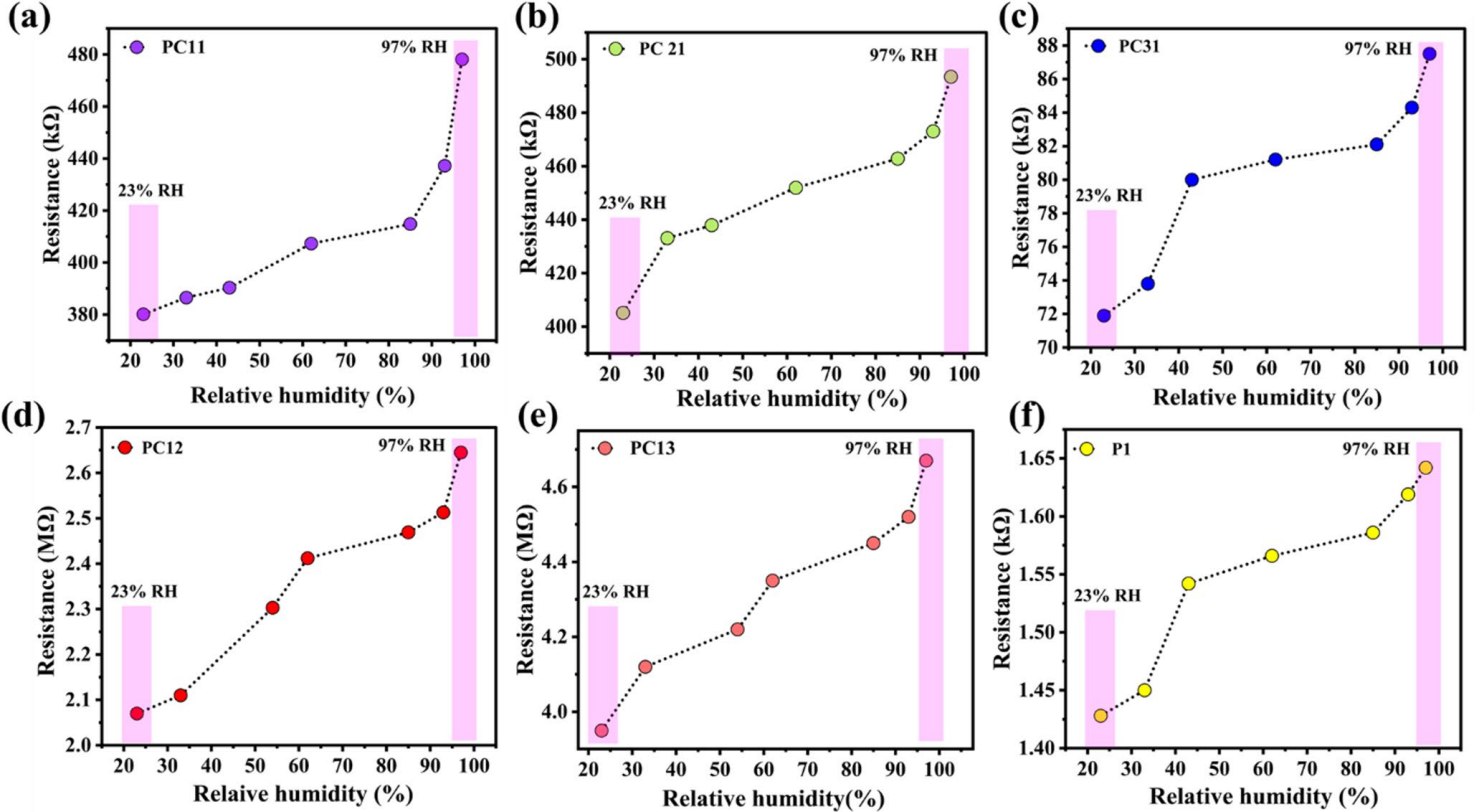
(**a**-**e**). Humidity sensing response of different PPy/CuO composite samples (PC11, PC21, PC31, PC12, and PC13) represented as resistance variation with respect to relative humidity ranging from 23% to 97%, (**f**) Response of pristine polypyrrole (P1), showing the baseline performance in comparison to the composite-based sensors

**Scheme 3:**
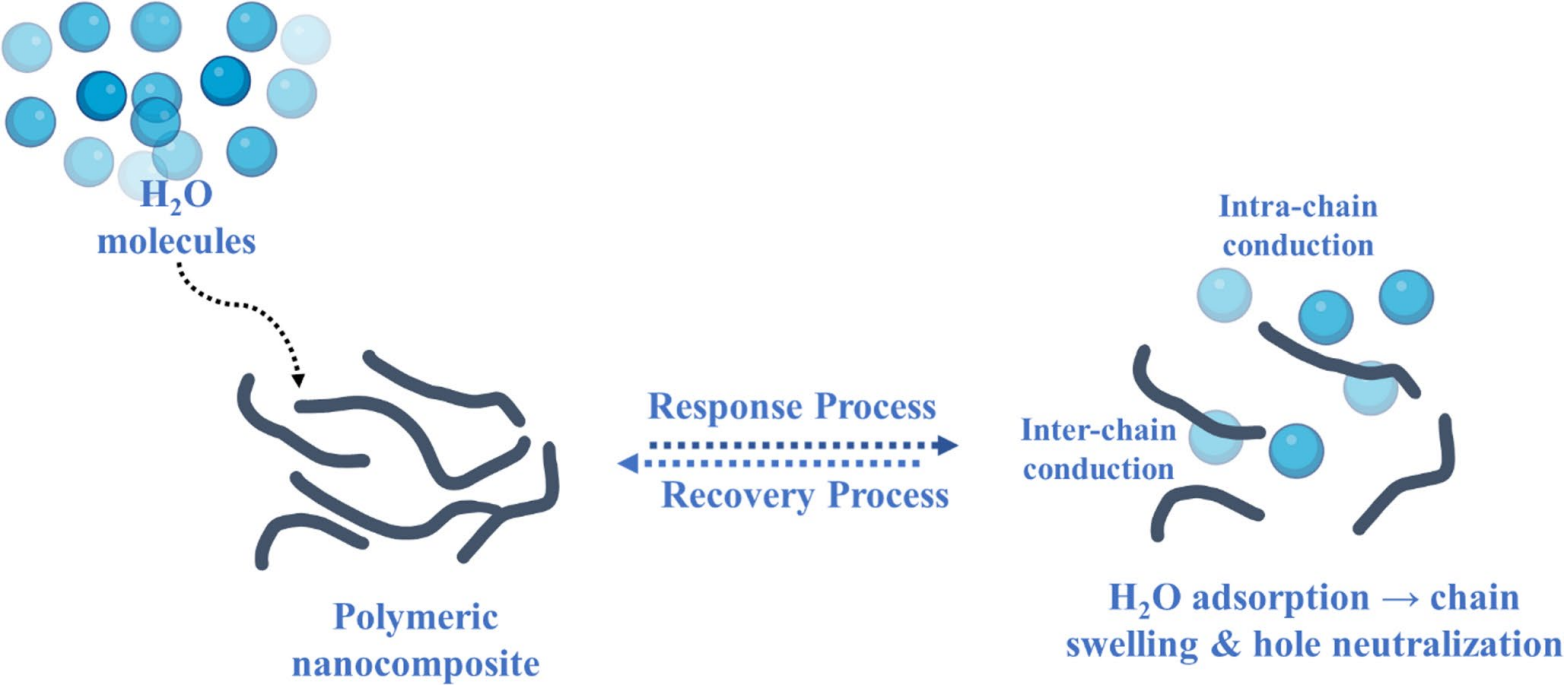
Shows the Schematic representation of the humidity sensing mechanism in the PPy/CuO nanocomposite, illustrating moisture-induced polymer swelling, modulation of intra- and inter-chain charge transport, and increased resistance with rising relative humidity

An increase in resistance was noticed with rising humidity levels. This response can be related to the hygroscopic nature of the polypyrrole/copper oxide nanocomposite, which undergoes swelling upon water intake. This result modifies the interchain spacing of the polymer and hence disrupting the ion transport pathways. At elevated humidity levels water molecules are expected to accumulate at the interface between adjacent polypyrrole chains forming a thin water layer. This layer mitigates the effective charge transfer across the interfaces leading to a rise in interfacial tension as shown in Fig. 6(a-e). A comprehensive and plausible mechanism explaining the observed increase in sensor with elevated relative humidity is presented in Scheme 3, which schematically illustrate the humidity sensing mechanism in PPy/CuO nanocomposite. The electrical conductivity of PPy originates from both intra and inter chain charge transport process. The inter chain conductivity is explained by the spacing between adjacent polymer chains, the extent of conjugation and the relative orientation of the chains. While intra chain conductivity is largely influenced by structural defects along the polymer backbone, the effective conjugation length, and the proton transfer process involving adsorbed water molecules. Although proton assisted transport through adsorbed water molecules may lead to an increase in charge conduction this effect is outweighed by the swelling induced interruption of the charge transport between the polymer chain, causing in an overall increase in resistance. Furthermore, PPy synthesised via chemical oxidative process acts as a p-type material with holes as the major charge carriers. The electrons provided by the adsorbed water molecules partially compensate these holes, leading to an additional increase in resistance with the rise in humidity. This characteristic is in good agreement with previously reported studies on humidity induced conductivity modulation in polypyrrole based materials. The observed higher resistance with increased CuO content under similar conditions can be ascribed to several interconnected factors. The CuO shows a much lower intrinsic electrical conductivity compared to polypyrrole, thus the overall conductivity declined as the proportion of the material increased in the nanocomposite. The higher loading of CuO disrupt the continuity of the conductive network laid by polypyrrole thereby disrupting the percolation pathways vital for the effective transport of charges. The expanded interfacial region between the polypyrrole and the metal oxide may also cause the elevated resistance as these interfaces likely serve as barrier to the charge mobility because of discrepancies in electronic structure or poor charge transfer across the interface [39, 50].

**Fig. 7:**
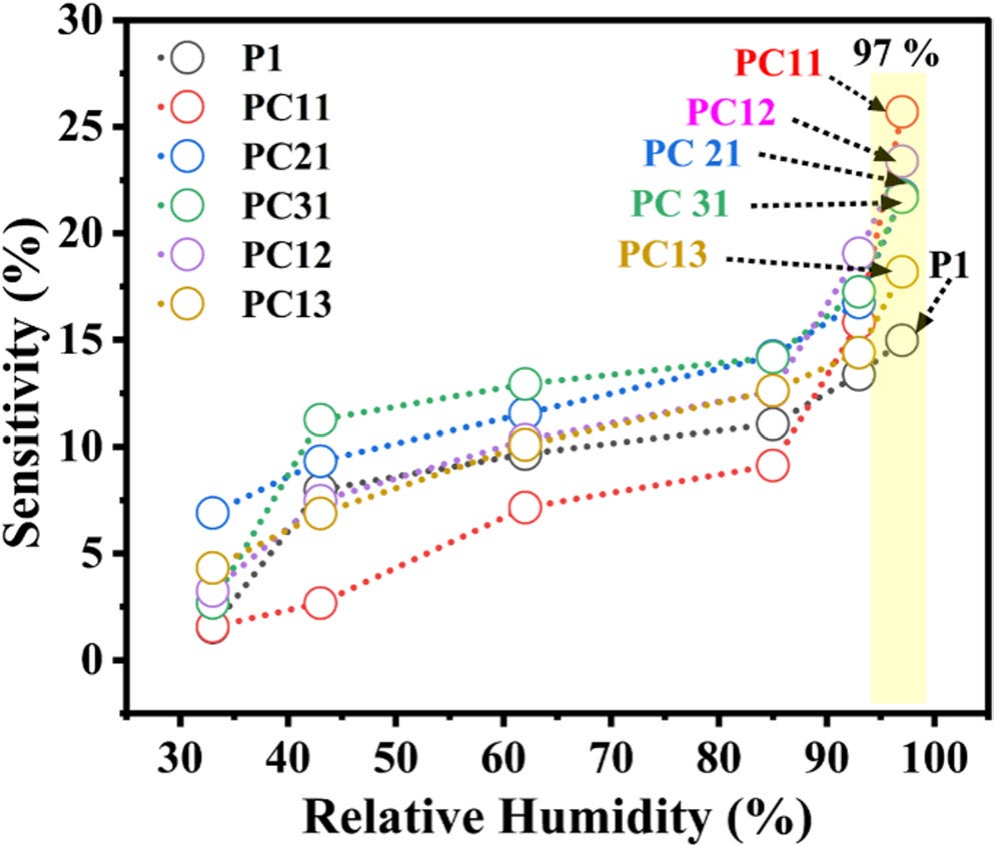
The Plot shows the sensitivity of the humidity sensor devices

The superior sensitivity of the 1:1 polypyrrole-copper oxide composite stems from several complementary mechanisms. Copper oxide nanoparticles possess inherent hydrophilic properties due to their surface hydroxyl groups and oxygen-rich structure, which facilitates strong interactions with water molecules through hydrogen bonding. When combined with polypyrrole, which also exhibits hydrophilic characteristics through its nitrogen-containing heterocyclic structure, the composite creates multiple active sites for moisture adsorption. The synergistic effect manifests in several ways: First, the copper oxide nanoparticles increase the overall surface area and porosity of the composite, providing more sites for water molecule adsorption. Second, the conductive polypyrrole matrix ensures efficient charge transport throughout the sensing material, enabling rapid electrical response to humidity changes. Third, the optimal 1:1 ratio creates an ideal balance where the copper oxide particles are well-dispersed within the polymer matrix without compromising the electrical continuity of the conductive network. Figure 7 shows the sensitivity curve.

### Response and recovery characteristics

**Fig. 8:**
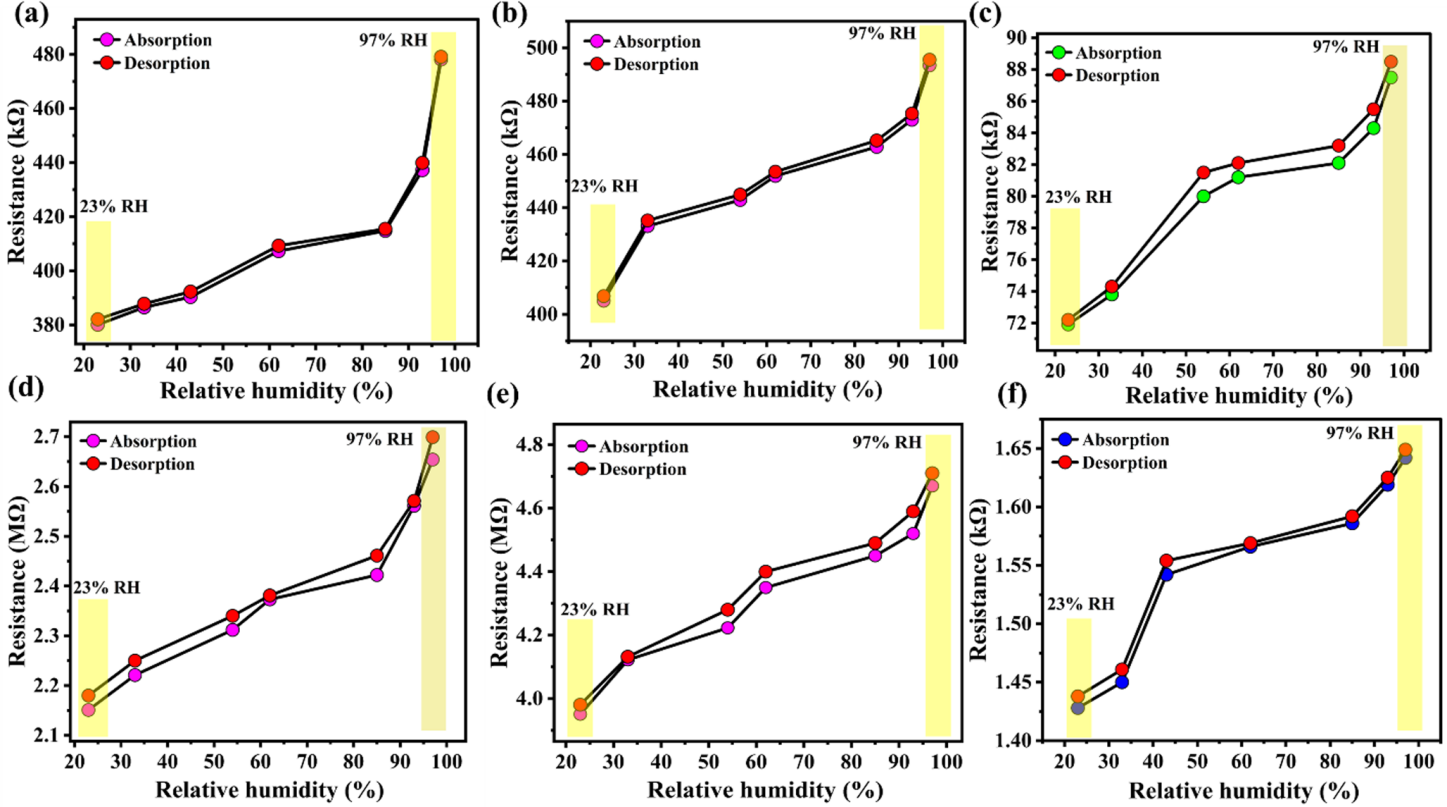
Hysteresis plots of resistance response versus relative humidity for (**a**) PC11, (**b**) PC21, (**c**) PC31, (**d**) PC12, (**e**) PC13, and (**f**) pristine polypyrrole (P1), showing absorption and desorption characteristics between 23% and 97% RH

Figure 8, which represents the Hysteresis behaviour of the fabricated humidity sensors, shows resistance response versus relative humidity (23–97% RH) for **(a)** PC11, **(b)** PC21, **(c)** PC31, **(d)** PC12, **(e)** PC13, and **(f)** pristine PPy (P1). A narrow loop was observed between the absorption and desorption branches, which reflects the excellent reversibility and stability of the sensor’s response under cyclic humidity exposure. The small lag in the desorption curve can be attributed to the slow release of physiosorbed water molecules, which temporarily remain trapped within the porous network and at the polymer/oxide interfaces. However, the overall low hysteresis suggests that the water molecules are not strongly chemisorbed into the polymer backbone but are instead weakly bound through hydrogen bonding or surface interactions, allowing for their rapid removal once the humidity decreases. This behaviour is further supported by the heterogeneous and porous morphology of the PPy/CuO nanocomposite, which provides interconnected pathways for water vapor diffusion and prevents deep penetration of moisture into the bulk material. Consequently, the facile adsorption/desorption dynamics not only ensure faster recovery but also enhance the long-term stability and repeatability of the device, which are essential features for reliable humidity sensing applications [51]. The hysteresis for PC11 nanocomposite shows better hysteresis in terms of adsorption and desorption across the relative humidity of 23% to 97% was as demonstrated in **(**Fig. 8a**)**, which outperforms the other counter nanocomposite and aligns a rapid response and recovery of the PC11 nanocomposite. Table 2 summarizes the hysteresis characteristics of the fabricated humidity sensor devices. The detailed Adsorption-desorption resistance and hysteresis of the fabricated humidity sensors are mentioned in the supporting information (S3).

**Table 2:** Hysteresis characteristics of the fabricated humidity sensor devices

Sensor denotation	$\:Max.\:\%\:hysteresis$	$\:Min.\:\%\:hysteresis$	$\:Avg.\:\%\:hysteresis$
P1	5.43	1.36	3.56
PC11	2.73	0.71	1.69
PC21	2.65	1.77	2.24
PC31	9.04	1.81	5.59
PC12	8.21	1.46	4.93
PC13	9.22	1.32	5.59

### Influence of overprinting on device performance

**Fig. 9:**
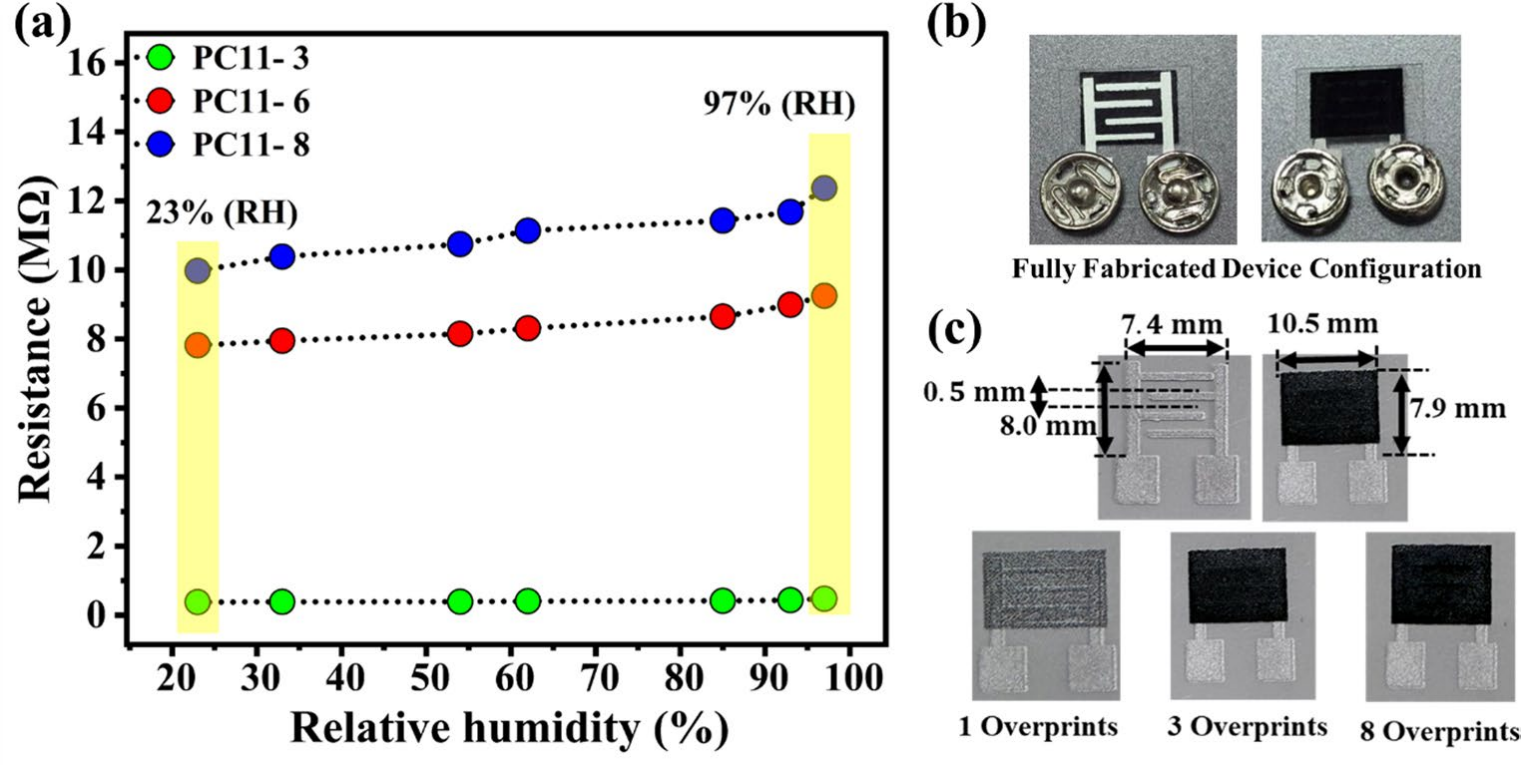
Variation in device performance with successive overprinting cycles, (**a**) Change in resistance of the PC11-based humidity sensor as a function of relative humidity (23–97% RH) for different overprinting cycles (PC11-3, PC11-6, and PC11-8), (**b**) Photographic images of the fully fabricated device, and (**c**) Dimensional representation of printed sensing layer and electrode configuration after overprinting

The plot illustrated in Fig. 9(a-c) shows the influence of overcoating on the polymer/CuO composite. An increase in the number of overcoating layers results in a corresponding rise in resistance, attributed to the fact that charge carriers must traversed a thicker medium. Consequently, the effective conduction pathways become longer and more tortuous, which enhances resistive behaviour. Furthermore, at the polymer–metal oxide interface, the adsorption of water molecules formed an insulating layer that hampered the charge transfer and diminished overall conductivity. This effect leads to the swelling of the composite in humid conditions, which disrupts the π-π stacking of the polymer chains and further restricts charge mobility. The fully fabricated device configuration, optimized for analysis, is shown in Fig. 9(b). For the three- and eight-layer overprinting processes shown in Fig. 9(c), each printed layer was thermally dried at 50 °C for 30 s, followed by a 30 s cooling period at room temperature before deposition of the subsequent layer. This procedure was applied consistently for both the silver current collector layer and the sensing material to ensure good interlayer adhesion and thickness uniformity. The thickness of the sensor layers was measured using a *Mitutoyo Absolute digital thickness gauge*. The PET substrate exhibited a thickness of 0.183 $\:mm$, while the printed silver current collector measured 0.008 $\:mm$. After three successive coatings, the thickness of the deposited sensing layer was 0.042 $\:mm$. In addition, long-term stability was assessed for the PC11 sensor over 15 consecutive days at 97% RH, during which the sensor exhibited only minimal deviation from the initial resistance response, with an error margin of approximately 0.01%, confirming excellent stability and repeatability. These additional analyses significantly strengthen the reliability and completeness of the humidity sensing evaluation presented in the supporting information, as Figure S1.

### Response and recovery time of the humidity sensor

**Fig. 10:**
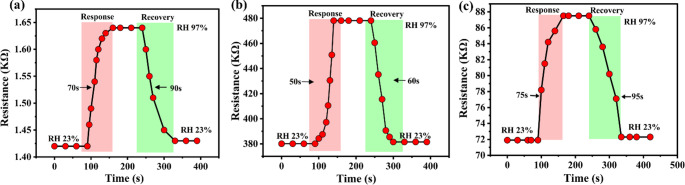
Response and recovery characteristics of the fabricated humidity sensors, (**a**) pristine PPy, (**b**) PC11, and (**c**) PC31, illustrating their adsorption/desorption behaviour and sensor reversibility

Figure 10(a-c) presents the response/recovery characteristics of pristine polypyrrole and the polypyrrole/copper oxide composite under varying relative humidity from the minima to maxima (23% to 97%). The curves confirmed the reversible behaviour of the sensors, with resistance increasing upon exposure to humid conditions and returning to the baseline as humidity decreases. Incorporation of copper oxide significantly reduced the response and recovery times to approximately 50 s and 60 s, respectively. Furthermore, dynamic response-recovery measurements were also performed under multiple relative humidity transitions from (23% to 54%) and (54% to 84%) as shown in supplementary as Fig. S1(a, b). which shows presents the real-time resistance variation of the sensor when exposed to stepwise changes in RH, demonstrating stable and reproducible sensing behaviour. At lower humidity variation (23% to 54% RH), the sensor exhibits a rapid response with a response time of approximately 20 s, followed by a recovery time of 27 s upon restoring the initial humidity level. Similarly, at higher humidity variation (54% to 85% RH), the response and recovery times are reduced to ~ 18 s and ~ 25 s, respectively. The reduction in response time was attributed to the higher density of active sites introduced by the oxide and the enhanced porosity of the composite, which speeds up the diffusion of water molecules. The shorter recovery time arose from the limited penetration of water into the bulk of the material and the predominance of physisorption, aiding the easier desorption [52]. Overall, these findings highlight the improved kinetics of the polypyrrole/copper oxide composite, while also confirming the effectiveness of polypyrrole as a humidity-sensitive material in the 23–97% RH range, demonstrating good sensitivity, reversibility, and stable performance [53].

## Real-time sensing behaviour

**Fig. 11:**
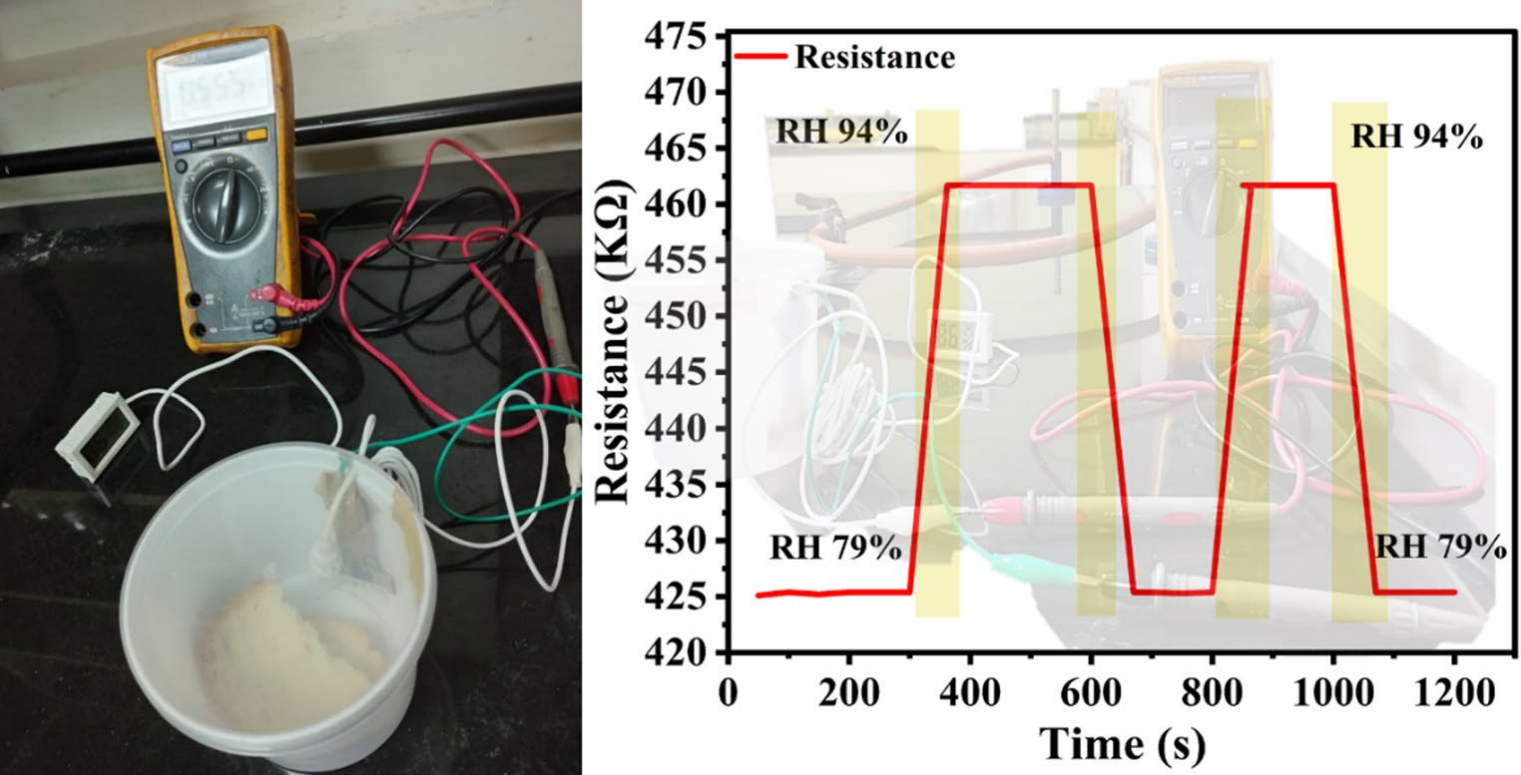
Real-time analysis of the device response under dynamic conditions, demonstrating the stability and reproducibility of sensing performance

The real-time applicability of the fabricated humidity sensor was demonstrated using a moist bread loaf as a natural humidity source, as shown in Fig. 11, where the relative humidity (RH) increased up to 94%, while the ambient room humidity served as the lower reference, monitored simultaneously with a hygrometer. The PC11-based sensor exhibited rapid and reversible changes in resistance, with response and recovery times in close agreement with those obtained under controlled RH conditions using saturated salt solutions, confirming the reproducibility and reliability of the device. Notably, the PC11 composition outperformed pristine PPy by delivering faster response kinetics and higher sensitivity, owing to its porous morphology and the synergistic role of CuO in enhancing water adsorption/desorption dynamics. Long-term stability analysis further revealed that the sensor maintained nearly constant humidity readings for seven consecutive days with only negligible fluctuations, highlighting its robustness. These results collectively demonstrate that the PPy/CuO composite sensor, particularly the PC11 formulation, is well-suited for practical real-time humidity monitoring in diverse environments [54, 55]. Furthermore, Table 3 presents a comparative analysis of the present work with those reported in the previous literature. In addition, the long-term stability of the PC11 sensor was evaluated over 15 consecutive days at 97% RH. The sensor exhibited only a negligible deviation (~ 0.01%) from its initial resistance response, confirming excellent stability and repeatability, as shown in the Supplementary Information as Fig. S2.

**Table 3:** Comparison of the present work with similar other works

Sensing material	Method	Sensor Fabrication	Humidity Range	Response and Recovery	Ref.
Polypyrrole/Graphene	-	Two-beam laser interference	11–97%	149 s,150s	[56]
Polyaniline	Chemical oxidation	Spin deposition	10–97%	180 s, 60s	[36]
PPy/ZnO	Sonication	Solution dripping technique	10–97%	180 s, 60s	[27]
PPy/TiO_2_	Photo polymerization	UV-induced polymerization	30–84%	40 s, 20s	[57]
PPy/1,4-bromobutane	Chemical polymerization	Coating technique	10–97%	41 s,120s	[58]
PPy/CuO	Mechanical mixing	Screen printing	22–97%	50 s, 60s	Present study

## Conclusion

This study reports the formulation of a screen-printable PPy/CuO conductive ink using DMF as the solvent and CAP as the binder, optimized for humidity sensing applications. Unlike conventional studies focused solely on material synthesis or device performance, this work integrates ink formulation, printability, and sensing behaviour, addressing a key gap in scalable, low-cost polymer/metal oxide sensor fabrication. The enhanced sensing performance. among the tested compositions, the PPy/CuO nanocomposite with a 1:1 weight ratio, fabricated using screen printing technology, exhibited the most favourable sensing characteristics, including high sensitivity over a wide humidity range of 22–97% RH, fast response and recovery times of ~ 50 s and **~** 60 s, respectively, and minimal hysteresis. The p-p interfacial coupling between PPy and CuO enhances charge modulation at the composite interface, leading to improved sensitivity over a wide humidity range. Overall, this work introduces a stable and screen-printable p-type PPy/CuO composite ink, elucidates the role of p-p interactions in moisture sensing, and reports an inverse response mechanism, offering valuable insights for the development of robust, scalable, and high-performance humidity sensors for practical applications. Future efforts will focus on enhancing sensitivity through the incorporation of hydrophilic dopants such as polyethylene glycol (PEG), ethyl hydroxycellulose (EHC), and polyvinylpyrrolidone (PVP), as well as carbon-based nanomaterials, while maintaining ink stability and rheological integrity.

## Supplementary Information

Below is the link to the electronic supplementary material.

Supplementary Material 1 (DOCX 275 KB)

## Data Availability

Data will be made available on request.
