# Supplementary material for: Influence of CuO on the performance of conducting polymer matrix for screen-printed humidity sensing applications
Source: Mikrochim Acta. 2026 Feb 17;193(3):164. doi: 10.1007/s00604-026-07897-9 (PMC12913315; doi:10.1007/s00604-026-07897-9)
Supplement: Supplementary file 1 — Supplementary Material 1 (DOCX 275 KB) [file 604_2026_7897_MOESM1_ESM.docx]

**Influence of CuO on the Performance of Conducting Polymer Matrix for Screen-Printed Humidity Sensing Applications**

B S Manjunatha **^a^**, Shilpa Shetty **^a^**, Mohammad Saquib **^a^**, Suma A Rao **^a^**, Ramakrishna Nayak **^b^**, Vinod Kamath **^b^**, M. Selvakumar **^a^***

^a^ Department of Chemistry, Manipal Institute of Technology, Manipal Academy of Higher Education, Manipal 576104, Karnataka, India

^b^ Department of Humanities and Management, Manipal Institute of Technology, Manipal Academy of Higher Education, Manipal, 576104, Karnataka, India

Correspondence author*: M Selvakumar ([selva.kumar@manipal.edu](mailto:selva.kumar@manipal.edu))

1. **FTIR Analysis**

**Table S1. Tabulated comparative analysis of the wavenumber for the pristine materials.**

| **Wavenumber (cm^-1^)** | **Assignment** | **Inference** |
| --- | --- | --- |
| 2792,3067 | the C−H aromatic stretching |  |
| 1537 | C-C and C=C stretching of pyrrole ring | reveals conjugated backbone |
| 1286 | C−N stretching | Presence of nitrogen |
| 1447 | C–C stretching or ring vibrations within the pyrrole | combined C–C/C–N skeletal vibrations |
| 1015 | C–H in-plane deformation | Aromatic C–H bending |
| 765 | C–H out-of-plane deformation |  |

**Table S2. Tabulated comparative analysis of wavenumbers for the nanocomposite.**

| **Wavenumber (cm^-1^)** | **Assignment** | **Inference** |
| --- | --- | --- |
| 3369 | N-H stretching |  |
| 1542 | C=C stretching (pyrrole ring) | Intensity change in composite |
| 1274 | C−N stretching | Presence of nitrogen |
| 1450 | C–C stretching or ring vibrations within the pyrrole | combined C–C/C–N skeletal vibrations |
| 1031 | C–H in-plane deformation | Aromatic C–H bending |
| 778 | C–H out-of-plane deformation |  |
| 597 | Cu-O stretching vibration |  |

1. **Hysteresis Analysis**

**Table S3.** Adsorption-desorption resistance and hysteresis of fabricated humidity sensors.

| **% RH** | **AD (Resistance)** $\boldsymbol{(k\Omega)}$ | **DE** $\boldsymbol{(k\Omega)}$ | **Hysteresis** $\boldsymbol{(k\Omega)}$ |
| --- | --- | --- | --- |
| **Sensor PC11** | | | |
| 23 | 380.1 $\boldsymbol{k\Omega}$ | 382.1$\boldsymbol{k\Omega}$ | 2 $\boldsymbol{k\Omega}$ |
| 33 | 386.5 | 387.8 | 1.3 |
| 43 | 390.3 | 392.3 | 2 |
| 62 | 407.3 | 409.3 | 2 |
| 85 | 414.8 | 415.5 | 0.7 |
| 93 | 437.2 | 439.9 | 2.7 |
| 97 | 478.1 | 479.1 | 1 |
|  | **Fsv = 99** $\boldsymbol{k\Omega}$ |  | **Avg = 1.671** $\boldsymbol{k\Omega}$ |
| **Sensor PC21** | | | |
| 23 | 405.1 $\boldsymbol{k\Omega}$ | 406.8 $\boldsymbol{k\Omega}$ | 1.7 $\boldsymbol{k\Omega}$ |
| 33 | 433.1 | 435.2 | 2.1 |
| 54 | 442.9 | 444.9 | 2 |
| 62 | 451.9 | 453.5 | 1.6 |
| 85 | 462.8 | 465.2 | 2.4 |
| 93 | 473 | 475.3 | 2.3 |
| 97 | 493.4 | 495.5 | 2.1 |
| **PC21** | **Fsv = 90.4** $\boldsymbol{k\Omega}$ |  | **Avg = 2.0286** $\boldsymbol{k\Omega}$ |
| **Sensor PC31** | | | |
| 23 | 71.9 $\boldsymbol{k\Omega}$ | 72.2 $\boldsymbol{k\Omega}$ | 0.3 $\boldsymbol{k\Omega}$ |
| 33 | 73.8 | 74.3 | 0.5 |
| 54 | 80 | 81.5 | 1.5 |
| 62 | 81.2 | 82.1 | 0.9 |
| 85 | 82.1 | 83.2 | 1.1 |
| 93 | 84.3 | 85.5 | 1.2 |
| 97 | 87.5 | 88.5 | 1 |
|  | **Fsv = 16.6** $\boldsymbol{k\Omega}$ |  | **Avg = 0.9286** $\boldsymbol{k\Omega}$ |
| **Sensor PC12** | | | |
| 23 | 2.151 $\boldsymbol{M\Omega}$ | 2.18 $\boldsymbol{M\Omega}$ | 0.029 $\boldsymbol{M\Omega}$ |
| 33 | 2.221 | 2.25 | 0.029 |
| 54 | 2.312 | 2.34 | 0.028 |
| 62 | 2.373 | 2.381 | 0.008 |
| 85 | 2.422 | 2.461 | 0.039 |
| 93 | 2.561 | 2.571 | 0.01 |
| 97 | 2.654 | 2.699 | 0.045 |
|  | **Fsv = 0.548** $\boldsymbol{M\Omega}$ |  | **Avg = 0.02686** $\boldsymbol{M\Omega}$ |
| **Sensor PC13** | | | |
| 23 | 3.951 $\boldsymbol{M\Omega}$ | 3.981 $\boldsymbol{M\Omega}$ | 0.03 $\boldsymbol{M\Omega}$ |
| 33 | 4.122 | 4.132 | 0.01 |
| 54 | 4.223 | 4.28 | 0.057 |
| 62 | 4.35 | 4.4 | 0.05 |
| 85 | 4.45 | 4.49 | 0.04 |
| 93 | 4.52 | 4.59 | 0.07 |
| 97 | 4.67 | 4.71 | 0.04 |
|  | **Fsv = 0.759** $\boldsymbol{M\Omega}$ |  | **Avg = 0.04238** $\boldsymbol{M\Omega}$ |
| **Sensor P1** | | | |
| 23 | 1.428 $\boldsymbol{k\Omega}$ | 1.438 $\boldsymbol{k\Omega}$ | 0.01 $\boldsymbol{k\Omega}$ |
| 33 | 1.45 | 1.461 | 0.011 |
| 43 | 1.542 | 1.554 | 0.012 |
| 62 | 1.566 | 1.569 | 0.003 |
| 85 | 1.586 | 1.592 | 0.006 |
| 93 | 1.619 | 1.625 | 0.006 |
| 97 | 1.642 | 1.649 | 0.007 |
|  | **Fsv = 0.221**$\boldsymbol{k\Omega}$ |  | **Avg = 0.00786** $\boldsymbol{k\Omega}$ |

**Table S4.** Comparative humidity sensing performance of fabricated sensors

| **Sensor** | **Sensitivity** | **Avg. % hysteresis** | **Response time (s)*** | **Recovery time (s)** |
| --- | --- | --- | --- | --- |
| P1 | 14.9 | 3.56 | 70 | 90 |
| PC11 | **25.7** | **1.69** | **50** | **60** |
| PC21 | 21.8 | 2.24 | - | - |
| PC31 | 21.7 | 5.60 | 75 | 95 |
| PC12 | 23.4 | 4.90 | 62 | 89 |
| PC13 | 18.2 | 5.59 | - | - |


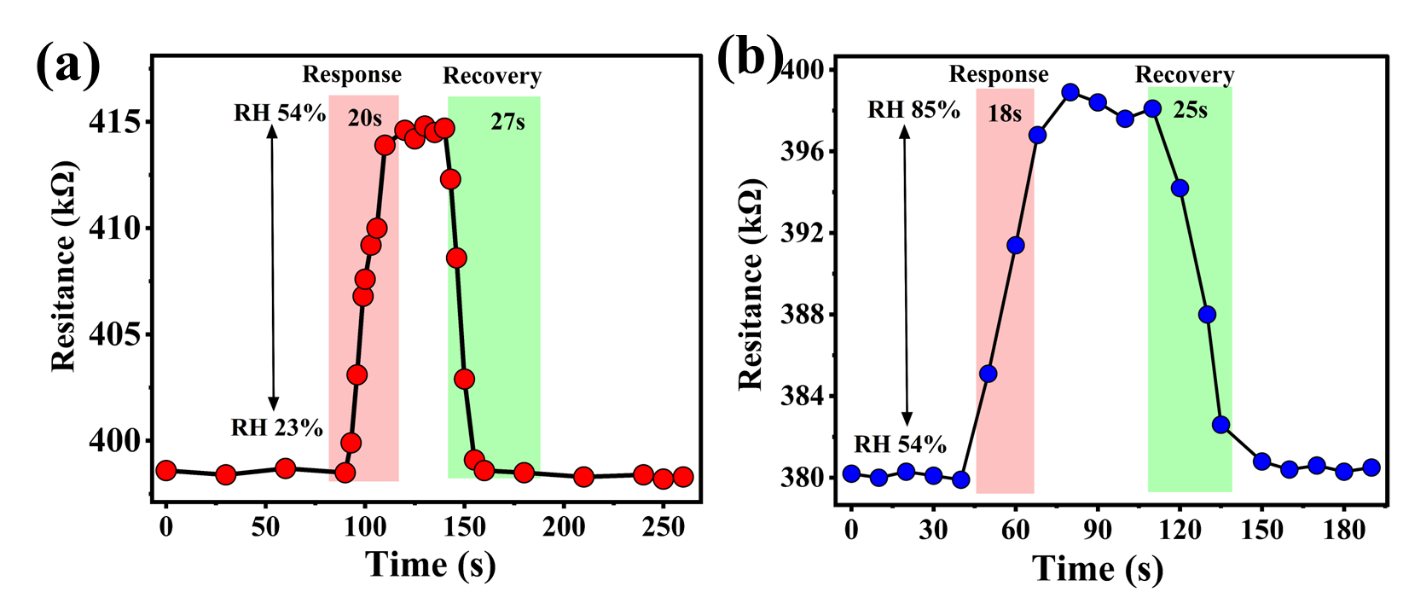


**Figure S1.** Dynamic response–recovery curves of the humidity sensor for RH changes: (a) 23% → 54% and (b) 54% → 85%.


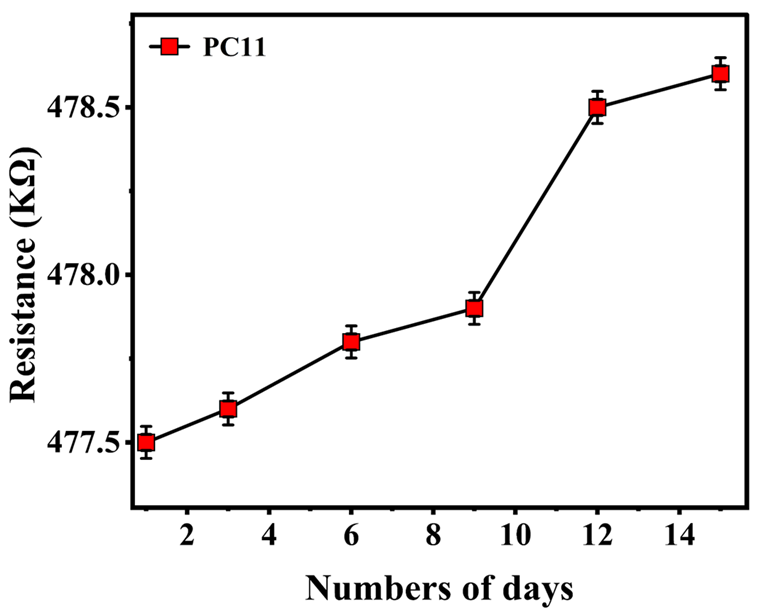


**Figure S2.** Long-term stability of the optimized PC11 humidity sensor was evaluated over 15 consecutive days at 97% relative humidity, showing minimal variation in resistance and excellent operational stability.
